# Process evaluation of the impact and acceptability of a polypill for prevention of cardiovascular disease

**DOI:** 10.1136/bmjopen-2015-008018

**Published:** 2015-09-30

**Authors:** Frances Wood, Abdul Salam, Kavita Singh, Sophie Day, Stephen Jan, Dorairaj Prabhakaran, Anthony Rodgers, Anushka Patel, Simon Thom, Helen Ward

**Affiliations:** 1 International Centre for Circulatory Health, Imperial College London and Imperial Healthcare NHS Trust, London, UK; 2 George Institute for Global Health, Hyderabad, Telangana, India; 3 The George Institute for Global Health, University of Sydney, Camperdown, New South Wales, Australia; 4 Centre for Chronic Disease Control and Center for Cardio-metabolic Risk Reduction in South Asia (CARRS), Public Health Foundation of India (PHFI), Gurgaon, Haryana, India; 5 Department of Endocrinology and Metabolism, All India Institute of Medical Sciences, New Delhi, India; 6 Patient Experience Research Centre, School of Public Health, Imperial College London, London, UK

**Keywords:** Cardiovascular Disease, Fixed Dose Combination, Medication Adherence, Polypill, Prevention

## Abstract

**Importance:**

The Use of a Multidrug Pill In Reducing cardiovascular Events (UMPIRE) trial has shown improved adherence with the use of a polypill strategy when compared with usual medications for cardiovascular disease (CVD) prevention. To advance from efficacy to impact, we need a better understanding of why and how such a strategy might be deployed in complex health systems.

**Objective:**

To understand, from the perspective of UMPIRE trial participants and professionals, how and why a polypill strategy improves adherence compared with usual care, why improvement is greater in some subgroups, and to explore the acceptability of a polypill strategy among trial participants and healthcare professionals.

**Design, setting and participants:**

A preplanned process evaluation, based on qualitative interviews, was conducted with a subsample of 102 trial participants and 41 healthcare professionals at the end of the UMPIRE trial in India and Europe.

**Results:**

Most patients contrasted the simplicity of the polypill with usual medications that they found complex and, for many in India, expensive. Patients with low baseline adherence struggled most with complex medication lists, and those without established disease described less motivation to adhere when compared with people who had already been diagnosed with CVD; people in the latter group had already undertaken self-directed measures to adhere to CVD preventive medicines prior to entering the trial. Taking medication was one of many adaptations described by patients; these included dietary changes, stopping smoking and maintaining exercise. Most patients liked the polypill strategy, although some participants and health professionals were concerned that it would provide less tailored therapy for individual needs.

**Conclusions:**

Adherence to treatment lists with multiple medications is complex and influenced by several factors. Simplifying medication by using a once-daily polypill is one approach to CVD prevention that may enhance adherence. Prescribers should also consider the wide variety of adjustments that individuals need to make to cope with daily medication.

**Strengths and limitations of this study**

- A strength of this study is that it included exploratory interviews with relatively large numbers of trial participants and professionals, using a standard interview topic guide.
- Interviews were conducted in diverse settings in the UK and India, with trial participants and health professionals.
- A limitation of this study is that trial participants and health professionals may have been inclined to speak positively about the trial. Various measures were taken to reduce this likelihood including confidentiality and anonymity.

## Introduction

The burden of cardiovascular disease (CVD) is a major global priority.^1^ Aspirin, statins and antihypertensive therapy are effective in reducing cardiovascular events and mortality. The combined use of these drugs is clearly indicated in people at high risk of CVD, especially those who have already suffered a myocardial infarction or stroke. The majority of people who could benefit from these drugs do not have access to them, and among those who do, adherence is poor.^2^ Four recent trials^3–6^ have reported the impact of a polypill strategy on adherence to recommended preventive medications in patients with established CVD (or at high risk of CVD) in different settings: the Use of a Multidrug Pill In Reducing cardiovascular Events (UMPIRE) trial in India and Europe,^3^ the Kanyini Guidelines Adherence with the Polypill (Kanyini-GAP) trial in Australia,^4^ the IMProving Adherence using Combination Therapy (IMPACT) trial in New Zealand^5^ and the Fixed-Dose Combination Drug for Secondary Cardiovascular Prevention (FOCUS) trial in Argentina, Brazil, Italy, Paraguay and Spain.^6^

Three of the trials (UMPIRE, Kanyini-GAP and IMPACT) used similar protocols under the umbrella of the Single Pill to Avert Cardiovascular Events (SPACE) collaboration.^7^ All four trials have shown that administration of medications as a polypill confers clinically important improvement in adherence when compared with usual care. These promising results add weight to the argument for the wider use of cardiovascular polypills as a preventive strategy, although there remain a number of key issues to address.^8^

The three SPACE trials showed greater benefit in people who were less adherent to cardiovascular medications at baseline; two trials found a greater benefit in people at high risk rather than those with established disease (UMPIRE and Kanyini-GAP) and in smokers (UMPIRE, and a non-significant trend in Kanyini-GAP); the IMPACT study found a larger effect in younger people. Polypill discontinuation rates ranged between 22% and 37% after 12–18 months of follow-up.

To improve our understanding of the potential strengths and limitations of the polypill strategy, we carried out a process evaluation of the UMPIRE trial. The trial design has previously been reported.^3^ ^9^ UMPIRE was a randomised, open-label, blinded end point clinical trial (clinicaltrials.gov: NCT01057537). It compared a polypill-based strategy with usual care in individuals in Europe and India with established CVD or at high risk of CVD. Participants randomised to the polypill strategy were prescribed one of two polypill formulations: V.1 (aspirin, 75 mg; simvastatin, 40 mg; lisinopril, 10 mg; and atenolol, 50 mg) or V.2 (aspirin, 75 mg; simvastatin, 40 mg; lisinopril, 10 mg; and hydrochlorothiazide, 12.5 mg). The polypill was taken once daily. Participants allocated to usual care continued with their usual cardiovascular preventive medications. Both arms of the UMPIRE trial showed improved adherence to indicated cardiovascular medication (aspirin, statin and ≥2 blood pressure lowering medicines) from baseline, together with corresponding reductions in blood pressure and low-density lipoprotein cholesterol^3^ with improvements significantly favouring the polypill group. By the end of the trial (median follow-up 15 months), 22% of participants in the polypill arm had stopped taking the pill.

Qualitative process evaluations are increasingly used in trials involving complex healthcare interventions to explore health professionals’ and patients’ views of the intervention, to understand components of an intervention and the way the impact varies among subgroups.^10^ Process evaluations are particularly useful in multisite trials where delivery and uptake of interventions may differ across the sites. Exploring the circumstances influencing improved adherence and clinical outcomes in the polypill treatment group will provide guidance on the clinical utility of this strategy and its potential scale-up.

This paper presents the findings of a preplanned^9^ process evaluation examining the barriers and facilitators to adherence within the two treatment groups and among subgroups in which the polypill strategy appeared to be particularly effective. Our aim is to understand key findings from the UMPIRE trial from the perspective of participants and professionals, namely:

- How and why a polypill strategy for CVD prevention improves adherence compared with usual care;
- Why the benefit is greatest in those who have lower adherence at baseline, those who have yet to have a cardiovascular event and smokers;
- Why a significant proportion of participants discontinued the polypill during the trial.

In addition, the process evaluation explores the acceptability of a polypill strategy with trial participants and healthcare professionals.

## Methods

### Study design and sample

INPUT (INterpreting the Processes of the UMPIRE Trial) is a process evaluation based on qualitative interviews of the UMPIRE trial participants and healthcare professionals at three trial coordinating centres: Hyderabad and Delhi (India) and London (UK). Interviews based on a predefined protocol were carried out at the end of the trial.^11^

In both countries, a range of health professionals were identified locally and invited to an interview. They were involved in the UMPIRE trial in some way, either as investigators or by supporting their patients’ participation. In addition, local health professionals working in CVD prevention were identified and invited to be interviewed. More Indian health professional staff members were included to reflect the large number of trial sites (28 in India compared with 3 in Europe).

For the process evaluation, trial participants of diverse ages, genders, length of trial participation and treatment arms were invited. These included some who had discontinued the polypill. Interviews were conducted at the final trial visit between February and July 2012; at this point, the UMPIRE trial results were yet unknown. To capture the diversity of the Indian trial sites, the participant interviews took place across a selection of six of these sites and were conducted in English or appropriate local languages. When interviewers were available, initially consecutive trial participants were invited for interview; later on sampling was focused on demographic groups under-represented in the sample.

Interviews were conducted by two researchers in the UK and two in India. All interviews were audio-recorded and transcribed. In India, the transcriptions were translated from local languages where necessary and the accuracy of the translation was verified by multilingual researchers. Data were analysed using the exploratory method of grounded theory.^12^ The methods are described in detail elsewhere.^11^ ASM and FW were responsible for the analysis, and it was supervised by HW, ST and SD. It was initially done separately in the UK and India, and then the two contexts were compared and contrasted. Nvivo V.9.0 was the data management software used for the analysis.

## Results

One hundred and two trial participants were interviewed, 50 from the UK and 52 from India; 7 and 2 participants, respectively, declined to take part in the interviews. Data saturation was reached at this point. The characteristics of the interviewed participants are described in table 1.

**Table 1:** Description of the interviewed trial participants in India and UK

	UK			India		
	Polypill	Usual care	Total	Polypill	Usual care	Total
Number of participants (%) [number Of discontinuing polypill]	25 (50) [9]	25 (50)	50	31 (60) [2]	21 (40)	52
Mean age in years [SD]	70 [9.4]	69 [11.8]	69 [10.6]	57 [10.5]	55 [11.5]	57 [10.9]
Male	20 (80%)	20 (80%)	40 (80%)	27 (87%)	20 (95%)	47 (90%)
Current smokers	2 (8%)	3 (12%)	5 (10%)	1 (3%)	0 (0%)	1 (2%)
Number of participants adherent at baseline (%)	17 (68%)	18 (72%)	35 (70%)	13 (42%)	12 (57%)	25 (48%)
Diabetes mellitus	8 (32%)	5 (20%)	13 (26%)	11 (35%)	5 (24%)	16 (31%)
Established CVD	17 (68%)	19 (76%)	36 (72%)	27 (87%)	17 (81%)	44 (85%)

CVD, cardiovascular disease.

Forty-two health professionals were also interviewed. The 26 Indian health professionals interviewed came from 11 separate sites and included: 16 cardiologists, 8 other physicians (1 neurologist, 1 endocrinologist, 6 general practitioners) and 2 research coordinators. The 15 UK health professionals interviewed included: 5 cardiologists, 1 neurologist, 1 elderly care physician, 2 general practitioners, 3 specialist nurses and 2 pharmacists. In the UK and India, respectively, 26 and 1 healthcare professionals declined participation or did not respond to the invitation.

**Figure 1:**
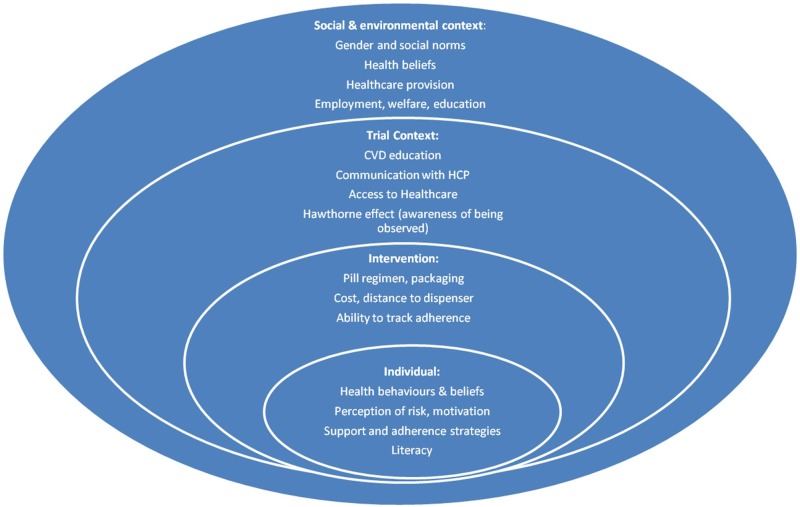
Model of medication adherence influences during the UMPIRE trial. The model demonstrates the abstracted themes that from the analysis of interviews were deemed influential to adherence in a trial context. This model may be useful for researchers designing future trials regarding adherence, because it outlines the multiple complex levels of influences on adherence that could be associated with a trial context. CVD, cardiovascular disease; HCP, healthcare professional; UMPIRE, Use of a Multidrug Pill In Reducing cardiovascular Events.

Interviews with trial participants and professionals revealed a wide range of influences on adherence in the context of the trial and more generally, many of which shed light on the overall results. A model of these factors is presented in figure 1, grouping them into individual, intervention, trial context and wider social and cultural levels. Table 2 lists a selection of quotes from the interviews highlighting the themes discussed below.

**Table 2:** Selection of quotes from the interviews

Quote 1	Obviously, convenience-wise, it[the polypill] was a lot easier because I think I take about six or seven tablets a day…and, obviously, with the polypill, it makes it a lot easier. So, there are only a few tablets to remember. [Trial participant, UK]
Quote 2	I have a bag with all my pills and…quite often, half way through taking my pills, I get disturbed, a phone might ring, my wife speaks to me, I then have to think, well did I actually take a pill from the package I'm holding…and I can't remember…with the polypill it wouldn't have mattered so much. It would have been less pills and it's much easier to know…because it's just a lovely packet, you can tell, normally when you have taken the pill or not. Because, you know, there's a big hole left if you have taken it, whereas with the other pills, you know I'm taking from different ends, I ignore the days on the back and things like that. [Trial participant, UK]
Quote 3	In my view, there is a small flaw in the trial, because the trial is looking for compliance and adherence. Any person who is given a study drug is likely to be more adherent, more compliant. Any person who is asked to continue the same old drug is likely to be less compliant or at least less interested in the trial. [Cardiologist, India]
Quote 4	He stopped[anticoagulants] for eight months…we told him…it doesn't cost much, maybe 5 rupees, 10 rupees. If you don't have money, you can skip your dinner or lunch, but don't skip this medicine. So sometimes we use very emphatic statements like that to tell the importance of these medicines. [Cardiologist, India]
Quote 5	When I came here for a check-up…they told me that there is going to be a lottery for drugs…There will be one capsule which combines 3–4 drugs and rest will be prescribed to me. It will reduce my expenses. They gave 5–6 months’ worth of drugs in one instalment and then for 5–6 months in the second…the biggest benefit is that I do a job and this saved me money. I used to buy medicines worth 1000 rupees from the market, but after this, I had to buy medicines worth only 300–400 rupees. [Trial participant, India]
Quote 6	I have come to recognize them over time. For example, one capsule has a red stripe; there is a white pill that I now know that I have to take again in the morning. There is a white capsule that I have to take in the night. [Trial participant, India]
Quote 7	“Then this is 1 tablet, that is 4, it is easier to have a single tablet…yes, I can't read, I used to recognize them…yes, by the colour. Sometimes I could make it out also…when I had to buy from the market…I took the medicine that I had to take in the evening, then had it again…this happened a couple of times. [Trial participant, India]
Quote 8	…if they've not had a heart attack then really they feel healthy. And most people don't like taking loads of things…[Cardiologist, UK].
Quote 9	If someone’s risks are that high, I think doctors will want to fine tune it[CVD medication] themselves…and if risks aren’t that high, I don’t think you’ll get long term compliance…with patients…until you have made it clear that this is a long term strategy, there's no point…they go away and take it and think, oh I don’t want to. So I think…taking time to start people on medication is really, really important…understanding what it’s all about. So people aren’t afraid that they’re turning into grandma by taking tablets at 40 years old. [General Practitioner, UK]
Quote 10	…it's an opportunity for me to be monitored if you like because I suppose when I first had the heart attack I was obviously in hospital then had some therapy afterwards. Once that was finished it was sort of “Here are your pills, off you go and get on with it”. You got all the advice and everything else and stuff like that. But then after a while I remember going to the doctor about something completely different but asked the question: “What happens now?” “Am I monitored yearly or what happens.” “Who looks after me?” That was initially soon after the heart attack. So I just thought it's[trial participation] a good opportunity to get an idea of how I'm doing but then it's also of some benefit to you guys. So it benefits both of us really. [Trial participant, UK]
Quote 11	I always believed before taking these that I would be the sort of person—because before having the heart attack for many years I was diagnosed as having high blood pressure, I was forgetful so I would sometimes go days without taking the pills. But since having the heart attack: no, I've become very regimented with it. So I don't think I've ever forgotten even if we go on holiday. I've got a pill box and it's all set out. [Trial participant, UK]
Quote 12	I don’t forget. It has become my daily routine for the last 1¾ years. I fear that if I don’t take the medicine, the pain will start again or I will become breathless. [Trial participant, India]
Quote 13	They may not be concerned about taking the medicine. This could be the reason. I start worrying if I don’t take my medicine. So, I don’t forget it. If they don’t feel pain, they become careless. [Trial participant, India]
Quote 14	I think these are all psychosocial problems. Anybody who is taking too much alcohol, too much smoking, something is wrong with his mind; his training has been negative or something. He knows that smoking is bad but still does it, they are less compliant. [Cardiologist, India]
Quote 15	I take a multivitamin when I’m working nights…well, you just don’t eat as well when you’re working the night shift…you have to rely on things like ready meals…believe me. Everybody says, “Oh, no, you could make a good dinner and take it with you.” No, no, no! It just doesn’t happen on nights…I work seven nights in a row and some of them are 12-hour shifts and when you go home, you’re tired. You’re not going to start doing that. [Trial Participant, UK]
Quote 16	The day there is tension I smoke. The day I give up smoking, God willing I will not need to have medicines. [Trial participant, India]
Quote 17	Well most of the research shows that roughly I think a third of patients…take it if at all…a third take it badly and a third take it correctly…the best compliance is with an informed patient and once daily medication with as fewer drugs as possible, so I can see the attraction of the polypill. [General practitioner, UK]
Quote 18	Sometimes I am having side effects because of your capsule…cough only…we will use only 25 every month, in that 25 also we will drop [skip] five [or] six, but it will stop. But when it stops I will continue again…[Trial Participant, India]

### Adherence is better with the polypill than with usual care

Overall, the improved adherence favoured the polypill, even in this trial population with relatively high baseline adherence.^3^ Adherence and motivation can be attributed to the convenience of a simplified regimen. This is best understood in relation to the challenges of the alternative polypharmacy. This convenience did not quantifiably improve adherence if participants were already competent adherers at baseline, but they uniformly reported that the polypill made adherence easier. The polypill was preferred over usual care by the majority of the participants who were interviewed (quote 1).

Convenience was attributed to the polypill in several ways including reduction in the number of pills taken per day, a decrease for some in the frequency of times a day pills were taken and fewer packages to transport and store (quote 2).

Adhering to the correct dose was made easier by the packaging of the polypill, which came in blister packs containing 1 week of tablets labelled with the days of the week. The polypill group had to return empty blister packs to the trial centre which may also have influenced reported adherence and made participants aware that others were observing their behaviour. The novelty of the polypill was also proposed as a positive influence on adherence (quote 3).

There was a cost differential between arms of the study for some participants, but overall in the UMPIRE trial there was no difference in adherence between those who were and were not exempt from medication or prescription charges.^3^ Nevertheless, interviews with individual participants and professionals in India highlighted cost as an important factor due to the significant expense incurred by some patients in the usual care group. In contrast, most UK participants were either exempt from prescription charges or on a fixed payment scheme for all their prescriptions. While some participants in India were exempt from charges through welfare or insurance systems, for others it was a major barrier. One health professional highlighted this, describing an encounter with a patient who had stopped taking anticoagulants after valve surgery (quote 4).

Free treatment was an incentive in some circumstances as well as a likely benefit of the polypill strategy itself (quote 5). Participants in India gave many examples of the difficult decisions they had to take if they were paying for multiple pills or other healthcare interventions.

### The polypill works best in people with poor adherence at baseline

The UMPIRE trial reported a higher impact in those who were less adherent at baseline. Such participants typically showed a >3-fold improvement in adherence if they were randomised to the polypill.^3^ This reflects the difficulties some people have with taking multiple medications; those who struggle are likely to benefit most from a simplified regimen.

Participants described how medications were sometimes missed, delayed, taken by accident, deliberately omitted or taken in excess. Multiple factors appeared to contribute to this including side effects, cost, changes in routine, travelling, tiredness, household roles, shift work, time constraints, forgetfulness, illiteracy and running out of supplies. Some participants indicated that more complex medication regimes increased the chances that they would diverge from their prescribed regimen. For people with such difficulties, and who are therefore less adherent at baseline, the polypill can have a greater impact than for those who are already highly adherent. Participants in the UK often expressed confidence that they never missed medication while using the polypill and emphasised their successful adherence by returning their empty blister packs at their scheduled visits.

UK participants discussed trying to overcome the complexity of their usual daily medicine regimes by developing personal strategies for adherence, for example, distributing pills in to a pill box, keeping a written record of what medications they had taken or keeping their medications on a visible surface to remind them. Additional strategies were reported in India. Many Indian participants were illiterate and described the challenge of having to identify pills. They often relied on support for identifying tablets or memorised the colour and shape of their medications. The polypill was sometimes described as larger than other tablets and harder to swallow but this feature also made it more distinctive (quotes 6 and 7).

### The polypill has greater adherence impact in those without established CVD

The UMPIRE trial found the polypill to have a greater impact in those at high risk of CVD than in those with established disease. Health professionals highlighted the issues of adherence for those at high risk (quote 8 and 9).

Most interviewees were highly motivated to take their medication, and those with established disease had less room for improvement as they had already developed effective strategies to take their multiple pills. In the UK, several patients described relatively subtle symptoms when they had been diagnosed with myocardial infarctions or other manifestations of vascular disease, and they were anxious about not recognising future events. This anxiety enhanced medication adherence and lifestyle modifications. It also motivated them to take part in research (quote 10).

Those UK participants with established CVD had attended education and cardiac rehabilitation programmes and often participated in the trial to have their health monitored (quote 11).

In India, the distinction between primary and secondary prevention was unclear in many interviews; some patients appeared to take their medications to alleviate symptoms rather than prevent future problems (quote 12).

There was variation in understanding of medication in both countries. Some participants could explain the purpose of each medication including the preventive role. Some believed that the polypill was for symptom relief, and others took the recommended medication without wanting to know the indication and placing trust in their doctor.

It is likely that the greater improvement observed in the trial in those without established disease compared with those with established disease was that there was less scope for the intervention to add to the higher underlying motivation in those with the experience of a cardiovascular event and diagnosis (quote 13).

### The polypill having greater adherence impact in smokers

The finding that the polypill had more of an impact in smokers was difficult to explore in detail as few smokers were recruited. Many participants had stopped smoking following a diagnosis of CVD. Overall, in the trial, 74% of ‘ever smokers’ were not current smokers.^3^ In the UK, interviewees who were current smokers appeared reluctant to discuss their smoking habits. In India, health professionals suggested that those who continued to smoke were unusual (quote 14).

A UK participant, who continued to smoke after having a stroke, described some of the structural difficulties he faced in adopting healthy behaviours. He talked of taking medication and vitamin supplements to make up for unhealthy lifestyle factors such as poor diet and work stress (quote 15). People have different strategies for managing their health such as uncertain future risks, and those who smoke may be more motivated to offset this risk by taking a preventive polypill. A patient from India indicated a belief that pill taking counteracted the negative effects of smoking (quote 16). Participants in both countries described other approaches to managing their health including homoeopathy, Ayurvedic therapy, changes in diet and exercise.

### Acceptability of a polypill strategy and reasons for discontinuation

In the UK and India, there was support from patients and some healthcare professionals for the polypill strategy due to its simplicity and lower cost (quote 17). However, one limitation to the polypill is the relatively high rate of discontinuation (quote 18). Discontinuation was a consequence of side effects or medical instruction,^3^ and partly reflected the limited variety of polypill versions available. In the UK, some participants who discontinued it said that if a polypill particularly suitable for them was available, they would prefer it to usual care. The polypill was not favoured by one participant in the usual care group who explained that she would not want to be on a polypill if it meant she was advised to take a higher dose of a medicine than was absolutely necessary.

Health professionals anticipated greater adherence through simplification of medication but had concerns about the perceived inflexibility of prescribing inherent to a polypill. Several commented that the components and doses in the available polypill version were not those recommended by the current guidelines. Many health professionals felt that they would need to be convinced by evidence that a polypill strategy was associated with better clinical outcomes than taking more personalised doses of a range of medications.

## Discussion

This paper describes the views and experiences of 102 UMPIRE trial participants and 41 health professionals in India and the UK to help interpret the trial findings and to understand why adherence favoured the polypill and specific subgroups. We also sought trial participants’ and health professionals’ views about the acceptability of a polypill strategy. The study used the exploratory method of grounded theory to interpret the interview data, leading to a proposed model of the influences on medicine adherence in the trial. This model can be taken into account when designing future interventions of this nature.

We have shown that the polypill is preferable primarily because usual care is complex and, for some groups, particularly in India, expensive. Participants’ descriptions of the challenges they faced with usual care involving multiple pills over the course of the day reveal the importance of simplified regimens. Strategies to aid adherence before the trial or for those in the usual care arm included the use of pill dispensers, record keeping and support from family members. Some groups manage the complex regimens better than others and this seems to explain the greater potential improvement with the polypill strategy for those who are not adherent at baseline. In some populations, high levels of illiteracy add to these challenges, with people struggling to remember instructions for pills of different size and colour at various times of the day. Unsurprisingly, those with lower motivation, including those who have not been diagnosed with a cardiovascular condition but rather told that they are at increased risk sometime in the future, are less likely to adhere to these complex regimens. The greater benefit in smokers and in those who had not yet had a cardiovascular event appears to be linked to complex motivations for including medication as part of a wider strategy for healthy living. We found many practical obstacles to adherence and a polypill appears to make it easier for some groups to overcome these.

This process evaluation revealed major differences between the process and context of the trial in the UK and India, even though the trial as a whole did not show a significant difference in impact on adherence between the two countries. In India, the free provision of medication in the polypill arm was a prominent influence described by participants and professionals in relation to improved adherence; in contrast, cost did not appear to be a major issue in the context of free or reduced price medicines within the UK National Health Service (NHS). The various reported reasons for adherence and non-adherence were consistent with factors reported in the literature^6^ ^13^ ^14^ including convenience through simplification of medication schedules and clear packaging.^6^ ^15^

We used exploratory interviews to look in detail at the context of the trial in each country, prior to knowing the trials result. Once the trial results were known, we then formulated research questions and further interpreted the interviews. Therefore, our process evaluation collected information about trial context at the time of the trial, without either the researcher or the participant being influenced by the trial results. However, not having the results when undertaking the interviews limited the ability to probe for specific results. Participants interviewed in India were on average younger than those interviewed in the UK; however, this reflects the differences in the UMPIRE trial populations and is reflective of the earlier onset of CVD in India compared with the UK.^16^ The strength of this evaluation is that it included interviews with relatively large numbers of trial participants and professionals, using a standard interview topic guide. There are limitations to this work. Participants in the UMPIRE trial, and those who took part in these interviews, had higher than average adherence at baseline adherence, but the generalisability of these findings on adherence to CVD prevention outside the context of a clinical trial setting is uncertain. Furthermore, the polypill group returned used blister packs to the trial centre, whereas the usual care group did not and this may have influenced participants’ behaviour.^17^ Including only one of the three European countries means the results are less generalisable, but focusing on one European site in greater depth allowed a more meaningful comparison with India. There were no systematic differences between those health professionals who participated, declined or failed to respond.

The process evaluation may have been subject to social desirability bias, with trial participants and health professionals being inclined to speak positively about the trial. Various measures were taken to reduce this likelihood; the study was identified as being funded separately and participants were reassured of confidentiality and anonymity, that there were no right or wrong answers and that all views were respected.

## Conclusion

This analysis shows that adherence to medication regimens is complex and influenced by a wide range of factors. Simplifying regimens through once-daily medication is one key part of improving adherence, but many other factors including access, cost and motivation of professionals and patients must also be addressed. Further studies of a polypill strategy for CVD prevention should ensure that data are collected on these broader factors, and the impact considered as part of a complex intervention to reduce the burden of non-communicable disease. In extending the use of a CVD polypill-based preventive strategy to other settings, policymakers should capitalise on its main strengths in terms of dosing convenience and potential advantage in terms of cost, by targeting its use in high-risk populations where the greatest gains can be achieved.
